# The impact of secondary school students’ perceptions of parental academic involvement and academic stress on internalizing problem behaviors: the mediating roles of psychological resilience and materialism

**DOI:** 10.3389/fpsyg.2025.1582493

**Published:** 2025-07-09

**Authors:** Li Xue

**Affiliations:** Department of Psychology, Beijing Normal University, Beijing, China

**Keywords:** perceived parental academic involvement, perceived parental academic stress, internalizing problem behaviors, international school middle school students, resilience, materialism

## Abstract

**Introduction:**

This study aimed to examine how perceived parental academic involvement and stress influence internalizing problem behaviors (i.e., depression and anxiety symptoms) among secondary school students in international schools, focusing on the mediating roles of psychological resilience and materialistic values.

**Method:**

A cross-sectional survey was conducted among 412 secondary school students (Mage = 16.28 years; 50.2% female) from international schools in Mainland China. Participants completed validated questionnaires assessing perceived parental academic involvement and stress, psychological resilience (CD-RISC-10), materialism (MVS), depression (PHQ-9), and anxiety (GAD-7). Hayes’ PROCESS macro 4.0 (Model 4) with bootstrapping (5,000 samples, 95% CI) was used to examine the parallel mediation effects. This population was selected due to the unique cultural and academic stressors faced by international school students, such as cross-cultural adjustment and elevated academic demands.

**Results and discussion:**

Perceived parental academic involvement negatively predicted internalizing problem behaviors, while perceived parental academic stress was positively associated with them. Psychological resilience and materialism both significantly mediated the relationship between parental academic stress and internalizing symptoms. However, only psychological resilience—not materialism—mediated the effect of parental academic involvement. Moreover, perceived parental involvement did not significantly predict adolescent materialism. The findings suggest that fostering psychological resilience and addressing materialistic values may help buffer the negative impact of academic stress on adolescents’ mental health. While the results provide valuable insights into the emotional adjustment of international school students, caution is advised in generalizing the findings to other student populations.

## 1 Introduction

Recent research has shown a growing concern for the mental well-being of secondary school students, particularly those in international schools. While students from economically advantaged backgrounds are often presumed to have better mental health outcomes (Devenish et al., 2017), internalizing problems—such as anxiety and depression—are increasingly reported among international school students (Wang et al., 2022; Pereira and Farah, 2023).

Unlike their peers in local school systems, international students often face a distinct combination of stressors: identity ambiguity due to frequent relocations, academic uncertainty linked to globally competitive university admissions, and cultural dissonance within multilingual environments (Choi and Park, 2023; Li and Tsai, 2023). A 2025 report found that 64% reported academic anxiety stemming from parental academic stress focused on elite university placements (Global Consortium for International Student Mental Health, 2025).

Family dynamics play a crucial role in shaping adolescents’ emotional health. Parental pressure, overinvolvement, and material reward strategies have been linked to heightened depressive symptoms and anxiety, particularly in high-achieving educational settings (Eriksen, 2021; Zhang et al., 2023; Liu et al., 2024). Recent findings suggest that materialistic values—such as associating self-worth with academic success and financial outcomes—mediate the relationship between parental expectations and internalizing symptoms (Ng et al., 2023). These values are often reinforced by competitive school environments that prioritize measurable achievements over psychological well-being (Barbayannis et al., 2022; Cho and Liang, 2022). Psychological resilience has emerged as a potential buffer against such stressors; however, when family expectations conflict with adolescents’ internal coping strategies, this resilience may be undermined (Yu et al., 2022).

However, few studies have explored how these family influences affect internalizing symptoms through underlying mechanisms in international schools. This study addresses this gap by examining the mediating roles of psychological resilience and materialistic values, both of which are increasingly relevant in globalized educational contexts (Yu et al., 2022; Ng et al., 2023).

### 1.1 The relationship between perceived parental academic involvement, perceived parental academic stress, and internalizing problem behaviors

Parental academic involvement—defined by Epstein (1987) as communication with educators and participation in school activities—is traditionally linked to reduced adolescent internalizing behaviors (Morris et al., 2010; Wickersham et al., 2020). While higher socioeconomic status (SES) families exhibit greater involvement (Li et al., 2020; Zhang et al., 2020), international schools present a paradox. Parents in these settings, despite high SES and resource investment (Liu and Zhang, 2023), often adopt performance-driven involvement—prioritizing elite university admissions over holistic development. A 2025 global study found 62% of these parents equate academic success with financial status, exacerbating student anxiety (Global Consortium for International Student Mental Health, 2025).

Crucially, involvement’s protective effects may reverse under high pressure. In Asian international schools, parental involvement combined with rigid monitoring (e.g., daily progress checks) predicts increased somatic complaints and emotional withdrawal, independent of SES (Zhao et al., 2024). Cultural mediators further explain this contradiction: parental involvement’s negative association with depression is weaker in Asian contexts (*β* = −0.09) than in Western settings (*β* = −0.18), reflecting collectivist achievement norms (Ng et al., 2023).

Notably, existing research has yet to adequately address international students and the nuanced dual role of parental involvement specifically in academic domains. We thus propose:

*Hypothesis 1a: Perceived parental academic involvement negatively predicts internalizing behaviors.*

Kaynak et al. (2023) define parental academic pressure from both behavioral and emotional perspectives. Behaviorally, it includes urging secondary school students to work harder or maintaining unrealistically high academic expectations that may surpass students’ capabilities. Emotionally, it manifests through controlling behaviors, punitive responses to failure, non-supportive feedback, and emotionally charged expectations for success. Such pressure can significantly influence both academic outcomes and psychological health (Pomerantz and Wang, 2009; Dan et al., 2021).

Recent studies have found a strong link between excessive parental academic pressure and internalizing symptoms such as depression, anxiety, and academic burnout (Arusha and Biswas, 2020; Quach et al., 2015; Liu et al., 2024). High parental expectations often lead to students’ academic disengagement and emotional withdrawal when their perceived abilities fall short of those expectations (Raufelder et al., 2015; Worku et al., 2020). Although parental involvement—especially among Chinese families—can promote academic engagement, it does not necessarily enhance students’ emotional well-being or resilience (Cheung and Pomerantz, 2015; Zhang et al., 2023).

In international school settings, parents are typically highly involved and often hold strong academic expectations. However, this involvement frequently translates into perceived academic pressure, particularly in culturally diverse and performance-driven environments (Nguyen et al., 2023; Pereira and Farah, 2023).

While previous research has emphasized the positive correlation between parental expectations and student achievement (Pinquart and Ebeling, 2020), few studies have addressed how parental academic pressure that exceed students’ capacities contribute to internalizing problem behaviors. Consequently, this research proposes:

*Hypothesis 1b*: Perceived parental academic stress positively predicts internalizing problem behaviors in secondary school students.

### 1.2 The mediating role of psychological resilience

Psychological resilience refers to an individual’s capacity to recover from stress or adversity and return to a stable or positive psychological state (Troy et al., 2023). As a key protective factor in adolescent development, resilience is positively associated with mental well-being and negatively linked to depression and anxiety (Bonanno et al., 2005; Kyriazos and Poga, 2024). Robinson et al. (2014) confirmed that resilience buffers the effects of depression and anxiety, while more recent findings further support its role in mitigating the impact of daily stressors in academic settings (Song et al., 2021; Kleinkorres et al., 2023).

Despite its well-documented benefits, the role of psychological resilience in the context of international secondary school students remains underexplored. Specifically, few empirical studies have examined how resilience functions as a mediating mechanism between family-related academic factors—such as perceived parental academic involvement and academic stress—and internalizing problem behaviors.

On one hand, high levels of perceived academic stress are consistently associated with lower psychological resilience among adolescents (Anagha and Navyashree, 2020; Aysel et al., 2019). Excessive academic pressure from parents may lead students to appear responsible and goal-oriented externally, while internally experiencing chronic frustration, anxiety, and emotional fatigue (Liu et al., 2024). When students internalize such pressure, their resilience may be weakened, increasing the likelihood of developing internalizing symptoms such as withdrawal, depression, or anxiety (Kaynak et al., 2023; Nguyen et al., 2023).

On the other hand, supportive parental academic involvement has been shown to foster adolescent resilience (Zimmer-Gembeck et al., 2023). Encouragement, emotional availability, and academic responsiveness from parents act as positive environmental resources that enhance a student’s ability to cope with stress (Masten and Reed, 2002; Zhang et al., 2023). Students who perceive their parents as engaged and supportive in their academic lives report stronger resilience and fewer mental health challenges (Forgatch and DeGarmo, 1999; Millerg et al., 2015). Such findings indicate that resilience may also mediate the beneficial pathway from academic involvement to lower internalizing behaviors.

Moreover, the resilience factor-process model (Kumpfer, 1999; Wadsworth and Compas, 2002) provides a useful framework for understanding these mechanisms. It posits that both protective and risk factors—such as parental involvement and academic stress—interact with internal coping mechanisms to shape developmental outcomes. In this view, perceived parental academic pressure is conceptualized as a risk factor, while perceived parental academic involvement functions as a protective factor influencing the development of resilience and subsequent psychological adjustment.

Accordingly, this study proposes the following hypotheses:

*Hypothesis 2a*: Psychological resilience mediates the relationship between perceived parental academic involvement and internalizing problem behaviors.

*Hypothesis 2b*: Psychological resilience mediates the relationship between perceived parental academic stress and internalizing problem behaviors.

### 1.3 The mediating role of materialism

Materialism refers to a value orientation in which the pursuit of material possessions and financial success is seen as a key life goal and a primary source of personal worth (Richins and Dawson, 1992). Studies consistently show that materialistic values are linked to lower life satisfaction (Shrum et al., 2022), decreased happiness (Belk, 1985), and higher rates of depression and anxiety (Kasser and Ryan, 1993; Dittmar and Isham, 2022). Kasser and Ahuvia (2002) further argued that when adolescents overly pursue wealth and status, it may result in both psychological and physical problems. Recent evidence shows that adolescents with high materialistic values are more prone to internalizing symptoms, particularly when they lack emotional support (Zawadzka et al., 2023; Kim and Lim, 2024).

Although prior research has paid limited attention to the pathways through which family factors influence adolescent materialism, emerging studies suggest that parental behaviors—particularly pressure and involvement in academics—may shape youth materialistic values. For instance, Kaynak et al. (2023) found that emotionally controlling parenting styles reinforce the belief that success is equated with external achievements like wealth or academic performance. Similarly, Flouri (2004) and Duh (2016) noted that parental pressure increases interpersonal insecurity, which in turn fosters materialistic orientations. In high-achieving contexts like international schools, where parental academic expectations are often intensified, students may adopt materialistic values as a coping mechanism to meet these expectations (Kleinkorres et al., 2023; Zhu and Kim, 2025).

At the same time, research also indicates that parental support—especially when it is emotionally responsive and academically encouraging—can reduce adolescents’ reliance on materialistic values. Chaplin and John (2010) found that emotionally supportive parenting was associated with lower levels of materialism among adolescents. This suggests that perceived parental academic involvement may act as a protective factor by reducing the likelihood that students adopt materialistic values as a way of coping with stress (Zimmer-Gembeck et al., 2023).

**Figure 1:**
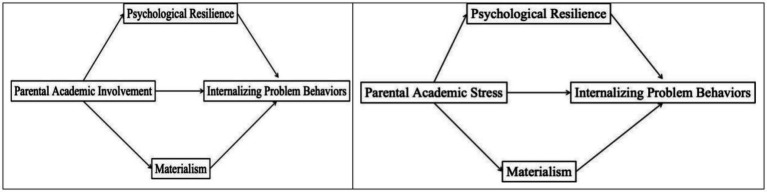
Theoretical hypothesized model.

Taken together, materialism may serve as a psychological mechanism through which family factors affect adolescents’ internalizing problem behaviors. Perceived academic pressure may increase materialism, while academic involvement may buffer against it (Figure 1). Therefore, this study proposes:

*Hypothesis 3a*: Materialism mediates the relationship between perceived parental academic involvement and internalizing problem behaviors.

*Hypothesis 3b*: Materialism mediates the relationship between perceived parental academic stress and internalizing problem behaviors.

## 2 Research methods

### 2.1 Participants and procedures

This study employed a random sampling method to recruit participants from an international school in Beijing. Specifically, all students enrolled in grades 10–12 were given an equal opportunity to participate. After obtaining approval from the their parents or other Guardian, students were invited to take part in the study voluntarily. Participation required signing an informed consent form, and the study protocol received approval from the institutional ethics committee.

Data collection was conducted in classroom settings. Students accessed the web-based questionnaire via a secure link and completed it individually on school-provided computers. Prior to the survey, homeroom teachers emphasized the importance of responding truthfully and based on personal experience to minimize social desirability bias.

A total of 433 responses were initially collected. To ensure data quality, 21 responses were excluded based on two criteria: (1) completion times under 200 s, which were considered insufficient for attentive reading and meaningful engagement with the content; and (2) highly repetitive or patterned answers indicating potential inattentive responding. These exclusion thresholds were determined based on prior research identifying minimum response times and answer variability as reliable indicators of data validity in self-report surveys (e.g., Leiner, 2019; Meade and Craig, 2012). After exclusion, 412 valid responses were retained, yielding a response rate of 95%.

While this high response rate is commendable, it is important to consider potential non-response bias. Students who opted not to participate might differ systematically in terms of stress levels, academic engagement, or psychological wellbeing. Although demographic information on non-respondents was not available due to privacy regulations, the high participation rate helps mitigate—but not fully eliminate—this concern.

The final sample included 205 males and 207 females, with an average age of 16.38 years (SD = 1.08). Among participants, 83.5% of fathers and 70.37% of mothers held at least a college degree. Additionally, 90% of students came from traditional family structures, and 63.35% reported annual household incomes exceeding one million yuan. Participants first provided demographic information, then completed a battery of validated scales assessing perceived parental academic stress, perceived parental academic involvement, depressive symptoms (PHQ-9), anxiety symptoms (GAD-7), resilience, materialistic values, and subjective socioeconomic status.

### 2.2 Measures

#### 2.2.1 Perceived Parental Academic Stress Scale

Adapted from Kaynak (2021), this scale comprises 20 items across three dimensions: psychological stress, psychological constraint, and excessive expectations, using a 5-point Likert scale (1 = strongly disagree, 5 = strongly agree). Higher scores indicate greater perceived parental academic stress. To ensure cultural relevance for Chinese international school students, the scale was translated and back-translated following Brislin (1980) procedure, and items were reviewed by three bilingual experts in educational psychology. Minor wording modifications were made to reflect culturally specific expressions of parental expectations in Chinese academic contexts. In this study, the scale demonstrated an internal consistency reliability of 0.89.

#### 2.2.2 Perceived Parental Academic Involvement Scale

Adapted from Cheung and Pomerantz (2011), this scale is divided into separate questionnaires for mothers and fathers, each containing 20 items, and utilizes a 5-point Likert scale (1 = strongly disagree, 5 = strongly agree). Higher scores indicate greater perceived parental involvement. This scale was previously validated in Chinese cultural contexts (e.g., Ng et al., 2014), and a pilot test (*n* = 30) with local international school students confirmed the clarity and relevance of the items. The internal consistency reliability for this scale was 0.92.

#### 2.2.3 Internalizing Problem Behaviors Scale

This scale includes the PHQ-9 Depression Assessment by Kroenke et al. (2001) and the GAD-7 Anxiety Assessment by Spitzer et al. (2006). The PHQ-9 consists of 9 items rated on a 4-point scale (1 = not at all, 4 = nearly every day), with higher scores indicating more severe depression. The internal consistency reliability of the PHQ-9 was 0.93. The GAD-7 includes 7 items, also rated on a 4-point scale, with higher scores indicating more severe anxiety. The internal consistency reliability of the GAD-7 in this study was 0.816. Both scales have been widely used and validated among Chinese adolescents (Zhou et al., 2020), supporting their applicability in this context.

#### 2.2.4 Resilience Scale

The Connor–Davidson Resilience Scale (CD-RISC) by Connor and Davidson (2003) consists of 25 items across three dimensions: tenacity, strength, and optimism, rated on a 5-point Likert scale (1 = never, 5 = always). The Chinese version of the CD-RISC developed by Yu and Zhang (2007) was used, which has demonstrated robust psychometric properties among Chinese youth populations. The internal consistency reliability in this study was 0.96.

#### 2.2.5 Materialism Values Scale

Adapted from Richins (2004) 9-item scale by Sun et al. (2020), this scale uses a 5-point Likert scale (1 = strongly disagree, 5 = strongly agree). Higher scores indicate greater levels of materialism. The adapted version has been previously validated with Chinese adolescents, showing good cultural fit and internal consistency (Sun et al., 2020). The internal consistency reliability of this scale in this study was 0.82.

#### 2.2.6 Control variable

##### 2.2.6.1 Subjective socioeconomic status

This variable was assessed using the second item from the Adolescent Subjective Social Status Scale by Hu et al. (2012), which was adapted to better reflect the perceived school-based social status among Chinese secondary school students. Cultural adaptation included replacing examples of Western social markers (e.g., private cars or extracurricular clubs) with locally relevant indicators (e.g., family education background and overseas travel frequency). It uses a 10-step ladder graphic (1 = lowest status, 10 = highest status) to measure perceived social status within the school environment. In the data analysis, subjective socioeconomic status was included as a covariate in all regression models to account for its potential confounding effect on internalizing problem behaviors. Specifically, it was entered as a continuous predictor in hierarchical multiple regression and mediation models using PROCESS Macro (Model 4), allowing for the isolation of its influence from the main variables of interest.

### 2.3 Data analysis

#### 2.3.1 Data analysis methodology

Data were entered and analyzed using SPSS 26 for descriptive statistics and correlation analysis. SPSS was selected due to its robust capabilities in handling large-scale survey data with minimal coding, making it particularly suitable for studies emphasizing psychometric scales and regression-based analyses. Compared with syntax-heavy platforms such as R or Mplus, SPSS offers a more accessible and transparent interface for implementing standard statistical procedures, facilitating replicability and clarity for applied psychological research.

Mediation analysis was conducted using the PROCESS 4.0 macro, Model 4, to examine parallel mediation effects. PROCESS macro, developed by Hayes (2017), is fully integrated within SPSS and is specifically designed for testing mediation and moderation models using ordinary least squares (OLS) regression. Its compatibility with the theoretical framework of this study—particularly the examination of parallel mediation pathways—makes it an optimal tool for hypothesis testing.

In the data analysis, subjective socioeconomic status was included as a covariate in all regression models to account for its potential confounding effect on internalizing problem behaviors. Specifically, it was entered as a continuous predictor in both the hierarchical multiple regression and PROCESS-based mediation models, allowing for the statistical isolation of its influence from the main variables of interest. All continuous variables were mean-centered prior to analysis to reduce multicollinearity.

## 3 Results

### 3.1 Common method bias

Since all data were derived from the self-reports of secondary school students, a Harman single-factor test was conducted to assess the potential for common method bias before analyzing mediation effects. The unrotated factor analysis revealed that 14 factors had eigenvalues greater than 1, and the first factor accounted for only 25.01% of the total variance, which is well below the 40% threshold commonly used to indicate significant bias. This indicates that common method bias was not a serious concern in this study (Zhou and Long, 2004).

### 3.2 Descriptive statistics and correlation analysis

As shown in Table 1, perceived parental academic involvement was significantly negatively correlated with perceived parental academic stress (*r* = −0.11, *p* < 0.05) and positively correlated with resilience (*r* = 0.32, *p* < 0.001). Additionally, it was negatively correlated with internalizing problem behaviors (*r* = −0.29, *p* < 0.001). These findings suggest that adolescents who perceive their parents as more academically supportive tend to experience lower levels of academic stress from parents, greater psychological resilience, and fewer internalizing symptoms such as anxiety or depression.

**Table 1:** Descriptive statistics and correlation matrix of variables.

Variable	*M*	SD	1	2	3	4	5	6	7
1.Gender	-	-	1						
2.Age	16.38	1.08	0.04	1					
3.Subjective socioeconomic status	5.54	2.50	0.01	−0.04	1				
4.Perceived parental academic involvement	3.76	0.83	0.01	−0.02	0.15^***^	1			
5.Perceived parental academic stress	2.38	1.00	0.03	−0.08	−0.09	−0.11^*^	1		
6.Resilience	3.69	0.75	0.08	0.05	0.23^***^	0.32^***^	−0.32^***^	1	
7.Materialism	2.95	0.75	−0.03	0.03	−0.06	−0.29^***^	0.28^***^	−0.12^***^	1
8.Internalizing problem behaviors	1.77	0.71	−0.07	0.05	−0.18^***^	0.29^***^	0.37^***^	−0.42^***^	0.30^***^

^*^p < 0.05, ^***^p < 0.001.

Perceived parental academic stress was negatively correlated with resilience (*r* = −0.32, *p* < 0.001) and positively correlated with internalizing problem behaviors (*r* = 0.37, *p* < 0.001). This indicates that higher levels of perceived parental stress may undermine adolescents’ adaptive capacities and increase their risk of internalizing emotional difficulties. Resilience was significantly negatively correlated with internalizing problem behaviors (*r* = −0.42, *p* < 0.001), while materialism was positively correlated with internalizing problem behaviors (*r* = 0.30, *p* < 0.001). This pattern reinforces the conceptual model in which resilience serves as a protective factor, whereas materialism may operate as a risk factor for internalizing problems.

In particular, gender and age were not significantly correlated with most of the primary variables, indicating that the observed effects are less likely to be confounded by these demographic factors. Similarly, subjective socioeconomic status was significantly related to resilience (*r* = 0.23, *p* < 0.001), internalizing behaviors (*r* = −0.18, *p* < 0.001), and parental involvement (*r* = 0.15, *p* < 0.001), and was thus controlled in the mediation models to enhance the robustness of the results.

### 3.3 Analysis of mediation effects

This study examined the parallel mediation effects of psychological resilience and materialism using Model 4 of the PROCESS 4.0 macro in SPSS. PROCESS was chosen for its ability to test complex mediation models with multiple mediators and covariates, providing estimates of total, direct, and indirect effects. The analysis used 5,000 bootstrap samples with a 95% confidence interval to test the significance of the mediation paths. Subjective socioeconomic status was included as a covariate to control for its potential confounding influence. This approach allows for a more precise estimation of how each mediator uniquely contributes to the association between perceived parenting variables and adolescents’ internalizing symptoms.

The model explored how perceived parental academic stress and involvement were related to internalizing problem behaviors through the mediating roles of resilience and materialism. Specifically, it tested whether parental stress heightened internalizing symptoms by weakening adolescents’ psychological resilience or promoting materialistic values, while parental involvement was hypothesized to reduce such symptoms by strengthening resilience and possibly lowering materialism.

#### 3.3.1 Parallel mediation effects of psychological resilience and materialism

After controlling for subjective socioeconomic status, a linear regression showed that perceived parental academic stress significantly predicted higher levels of internalizing problem behaviors (*β* = 0.21, *t* = 4.59, *p* < 0.001), supporting Hypothesis H1a. This indicates that adolescents who perceive higher academic stress from parents are more likely to exhibit symptoms such as anxiety, depression—suggesting a moderate, meaningful effect in practical terms.

**Figure 2:**
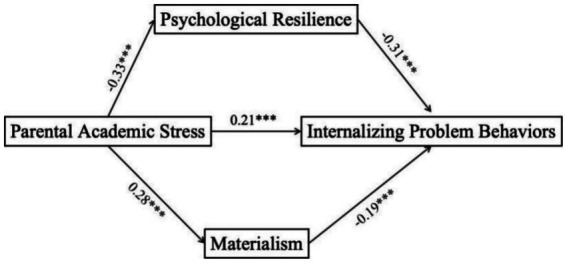
Parallel mediation effects of psychological resilience and materialism on perceived parental stress and internalizing problem behaviors. ^***^p < 0.001.

In the mediation model, perceived parental stress was negatively associated with psychological resilience (*β* = −0.30, *t* = −6.50, *p* < 0.001) and positively associated with materialism (*β* = 0.28, *t* = 5.82, *p* < 0.001). In turn, psychological resilience significantly predicted lower levels of internalizing problem behaviors (*β* = −0.31, *t* = −6.90, *p* < 0.001), while materialism predicted higher levels of such behaviors (*β* = 0.19, *t* = 4.40, *p* < 0.001), detailed in Figure 2. These findings suggest two distinct psychological pathways: parental stress may erode adolescents’ resilience, making them more vulnerable to internal distress, while also promoting materialistic values that, in turn, increase psychological burden.

A bias-corrected non-parametric percentile bootstrap method was used to test the significance of the multiple mediation effects. Using 5,000 bootstrap samples, the results confirmed both direct and indirect effects of perceived parental stress on internalizing problem behaviors.

As shown in Table 2, the total effect of perceived parental academic stress on internalizing problem behaviors was 0.36, with a 95% confidence interval of [0.27, 0.45], indicating a robust association. The direct effect remained significant (*β* = 0.21, *SE* = 0.05, 95% *CI* [0.12, 0.30]), accounting for 58% of the total effect. This suggests that more than half of the impact of parental stress on adolescents’ internalizing symptoms—such as anxiety or depression—cannot be explained by the mediators alone.

**Table 2:** Parallel mediation effects of psychological resilience and materialism on perceived parental stress and internalizing problem behaviors.

Path type	Path description	Effect	SE	95%CI	Relative effect percentage
Direct path	Perceived parental stress → Internalizing problem behaviors	0.21	0.05	[0.12, 0.30]	58%
Indirect path	Total mediation	0.14	0.05	[0.27, 0.45]	39%
	Perceived parental stress → Psychological resilience → Internalizing problem behaviors	0.09	0.02	[0.04, 0.16]	25%
	Perceived parental stress → Materialism → Internalizing problem behaviors	0.05	0.03	[0.02, 0.09]	14%

The indirect effects, transmitted through psychological resilience and materialism, jointly contributed to the remaining 39% of the effect (total indirect effect = 0.14). Specifically, the indirect pathway through psychological resilience had a stronger effect (*β* = 0.09, 95% *CI* [0.04, 0.16]), accounting for 25% of the total effect. This highlights the crucial protective role of resilience in buffering stress-related risks. The indirect effect through materialism was also significant, albeit smaller (*β* = 0.05, 95% *CI* [0.02, 0.09]), contributing 14% to the overall effect. This suggests that adolescents under academic stress may turn to materialistic values, which in turn are linked with heightened emotional distress.

**Figure 3:**
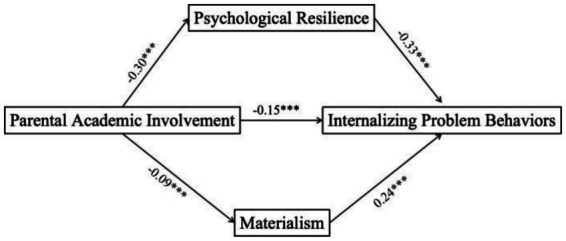
Parallel mediation effects of psychological resilience and materialism on perceived parental involvement and internalizing problem behaviors. ^***^p < 0.001.

Together, these findings underscore the dual psychological pathways through which perceived parental stress contributes to internalizing problem behaviors—both by diminishing adolescents’ internal coping resources and by promoting external value orientations. For further clarification, the detailed coefficients are presented in Table 2 and visualized in Figure 3.

#### 3.3.2 Parallel mediation effects of psychological resilience and materialism between perceived parental involvement and internalizing problem behaviors

A linear regression, controlling for subjective socioeconomic status, showed that perceived parental involvement significantly predicted fewer internalizing problem behaviors (*β* = −0.15, *t* = −3.39, *p* < 0.001). Mediation analysis revealed that perceived parental involvement positively predicted psychological resilience (*β* = 0.30, *t* = 6.48, *p* < 0.001), which in turn negatively predicted internalizing problem behaviors (*β* = −0.33, *t* = −7.10, *p* < 0.001). Although materialism positively predicted internalizing problem behaviors (*β* = 0.24, *t* = 5.50, *p* < 0.001), perceived parental involvement did not significantly predict materialism (*β* = −0.09, *t* = −1.81, *p* > 0.05).

A linear regression, controlling for subjective socioeconomic status, indicated that perceived parental academic involvement was significantly associated with lower levels of internalizing problem behaviors (*β* = −0.15, *t* = −3.39, *p* < 0.001). This suggests that adolescents who perceive their parents as more academically engaged tend to experience fewer symptoms such as anxiety, depression.

The mediation model further revealed that perceived parental involvement was positively related to psychological resilience (*β* = 0.30, *t* = 6.48, *p* < 0.001), which in turn predicted fewer internalizing problems (*β* = −0.33, *t* = −7.10, *p* < 0.001). This indirect pathway highlights the role of resilience as a key mechanism through which parental support buffers emotional difficulties.

Materialism, as expected, positively predicted internalizing problems (*β* = 0.24, *t* = 5.50, *p* < 0.001). However, the path from parental involvement to materialism was not statistically significant (*β* = −0.09, *t* = −1.81, *p* > 0.05). This may be because academic-oriented parental involvement fosters internal coping resources rather than shaping external value orientations. In summary, psychological resilience served as a significant mediator, whereas materialism did not. These findings suggest that while parental involvement promotes adolescents’ emotional adjustment primarily through resilience, its influence on materialistic values may be limited or indirect.

As shown in Table 3, perceived parental involvement had a significant total effect on internalizing problem behaviors (*β* = −0.27, 95% *CI* [−0.37, −0.18]). The direct effect remained strong and accounted for 78% of the total effect (*β* = −0.21, 95% *CI* [−0.30, −0.12]), indicating that higher parental involvement was associated with fewer internalizing symptoms such as anxiety and depression. This effect size suggests a meaningful impact, where even moderate increases in perceived parental support may reduce adolescents’ emotional distress.

**Table 3:** Parallel mediation effects of psychological resilience and materialism on perceived parental involvement and internalizing problem behaviors.

Path type	Path description	Effect	SE	95%CI	Relative effect percentage
Direct path	Perceived parental involvement → Internalizing problem behaviors	0.21	0.05	[−0.12, −0.30]	78%
Indirect path	Total mediation	−0.02	0.03	[−0.15, −0.06]	7.4%
	Perceived Parental involvement → Psychological resilience →Internalizing problem behaviors	−0.02	0.01	[−0.37, −0.18]	7.4%
	Perceived parental involvement → Materialism → Internalizing problem behaviors	0	0.02	[−0.05, 0.00]	0

Regarding mediation, psychological resilience significantly mediated the relationship between parental involvement and internalizing problems (*β* = −0.02, 95% *CI* [−0.15, −0.06]), accounting for 7.4% of the total effect. This suggests that supportive parental engagement may enhance adolescents’ psychological resilience, thereby reducing their vulnerability to emotional difficulties.

In contrast, the mediation pathway via materialism was not statistically significant (*β* = 0.00, 95% *CI* [−0.05, 0.00]). A possible explanation is that academic-related parental involvement may not directly shape adolescents’ materialistic values, which are more likely influenced by peer norms and societal messaging. When adolescents’ basic psychological needs—such as competence and relatedness—are met through parental support, they may be less inclined to seek validation through material means. By strengthening internal coping resources, parental involvement may buffer adolescents against the appeal of materialistic values as a means of emotional compensation.

Taken together, Table 3 highlights that the primary mechanism linking parental involvement to better mental health lies in bolstering internal coping capacities, rather than reducing external value orientations. Full mediation effects are visualized in Figure 3.

## 4 Discussion

### 4.1 Relationship between perceived parental involvement, academic pressure, and internalizing problem behaviors

This study demonstrates that perceived parental involvement is negatively correlated with internalizing problem behaviors, whereas perceived parental academic stress is positively correlated with these behaviors. These findings highlight the dual role of parental engagement in adolescents’ emotional development, depending on whether it is perceived as supportive or stressful.

Increased parental involvement generally results in fewer internalizing behaviors, a finding consistent with previous research (Mattingly et al., 2002; Graves and Wright, 2011; Li et al., 2018). This pattern was reaffirmed in a recent meta-analysis (Wang and Li, 2023), which found that warm, autonomy-supportive parental involvement enhances adolescents’ emotional regulation and reduces symptoms of depression and anxiety. This suggests that parental involvement serves a validating function, as greater parental support conveys to students that they are capable and valued, thereby enhancing their perceptions of competence and socio-emotional functioning (Grolnick and Slowiaczek, 1994). Studies have shown that students raised in supportive family environments are better equipped to manage academic stress and mitigate its adverse effects (Graves and Wright, 2011), indicating that parental support can alleviate academic stress and its negative outcomes.

Conversely, the study finds that higher perceived parental academic stress correlates with increased internalizing problem behaviors, a result that aligns with previous findings (Quach et al., 2015; Raufelder et al., 2015). This is further supported by recent cross-cultural research showing that academic stress perceived as parental pressure is strongly linked to emotional maladjustment among East Asian adolescents (Lee and Chao, 2024; Zhang et al., 2023). High parental expectations and academic pressures are perceptible to students and have a detrimental impact. Research indicates that perceived parental academic stress can lead to psychological control, where intrusive involvement converts into perceived stress, affecting students’ socio-emotional functioning and increasing the risk of anxiety and depression (Huntsinger et al., 2000). According to the Family Stress Model, perceived stress places individuals in a state of distress; greater parental support facilitates better adaptation, whereas a lack of support undermines social adaptation (Hill et al., 2004). Parental pressure and control not only harm the emotional well-being of parents themselves but also exacerbate the development of internalizing (e.g., depression and anxiety) and externalized problems in children (Manijeh, 2017).

The total effect of perceived parental academic stress (0.36) on internalizing problem behaviors is greater than that of perceived parental involvement (−0.27). This may be attributed to the unique characteristics of the study sample: participants from an international school in Beijing’s Haidian District, a highly competitive educational environment. Parents in such settings, often of higher educational and socioeconomic status, frequently impose significant expectations and pressures, which can excessively burden students. Previous studies have documented this phenomenon (Cheung and Pomerantz, 2011). Recent empirical evidence (He et al., 2024) shows that academic pressure in competitive urban schooling contexts predicts up to 40% of variance in adolescent depressive symptoms, underlining the gravity of this issue.

Additionally, during the critical period of junior and senior high school, intense academic competition exacerbates students’ stress, a key factor in internalizing problem behaviors (Córdova et al., 2023; Barbayannis et al., 2022). Increased parental academic pressure can further exacerbate these behaviors. Although Confucian philosophy emphasizes diligence and academic achievement, its influence can be ambivalent. Recent cross-cultural work (Yamamoto et al., 2025) highlights how Confucian ideals, while promoting effort and respect, may also reinforce internalized academic pressure when interpreted rigidly. Moreover, while parental involvement indicates greater participation, it does not necessarily convey positive emotional functions, consistent with Confucian philosophy that emphasizes continuous self-improvement (Li, 2002, 2005). Excessive contentment may diminish motivational drive. Thus, the cultural framing of parental behavior plays a critical role in determining its psychological impact.

In sum, this study contributes to the growing literature by disentangling the nuanced effects of parental behaviors on adolescent internalizing symptoms, especially within a culturally specific, high-stakes educational context.

### 4.2 The mediating role of psychological resilience

This study demonstrates that psychological resilience mediates the relationships among perceived parental involvement, perceived parental academic stress, and internalizing problem behaviors. Perceived parental involvement positively predicts psychological resilience, leading to fewer internalizing problem behaviors. Conversely, higher perceived parental academic stress undermines psychological resilience, thereby increasing internalizing problem behaviors. These findings align with existing research showing that perceived parental involvement and responsiveness are positively correlated with students’ psychological resilience (Forgatch and DeGarmo, 1999; Masten and Reed, 2002). Emotional warmth and positive support from parents enhance resilience levels (Miller et al., 2015), whereas perceived academic stress is negatively correlated with psychological resilience (Aysel et al., 2019). Recent evidence further confirms these associations: Wang et al. (2024) found that emotionally supportive parenting significantly predicts resilience and lowers adolescents’ risk for anxiety and depression in high-pressure school environments.

According to Kumpfer’s (1999) resilience framework model, individuals mobilize internal and external resources to cope with adversity. Psychological resilience, as a coping resource, is closely linked to academic pressure. When academic pressures become overwhelming, resilience must mobilize additional resources to counteract stress. Individuals with high resilience can marshal more resources and adapt effectively, whereas those with low resilience may struggle, disrupting their equilibrium. This process may involve changes in cognitive patterns, such as worldview and belief systems.

### 4.3 The mediating role of materialism

This study demonstrates that materialism mediates the relationship between perceived parental academic stress and internalizing problem behaviors, suggesting that higher perceived parental academic stress increases materialistic tendencies, which in turn exacerbate internalizing problem behaviors. This finding aligns with prior research indicating that materialism can serve as a stress-coping mechanism among adolescents, often used for affect regulation: under high stress, individuals are more likely to adopt materialistic values or engage in impulsive consumption to alleviate negative emotions (Roberts, 2012; Kaur and Singh, 2023). Recent studies have also highlighted that materialism is linked to reduced self-control and increased emotional dysregulation in high-pressure educational settings (Chen et al., 2024).

However, the results show that perceived parental involvement does not significantly predict materialism, which differs from studies showing that higher parental emotional care is associated with lower materialism in children (Richins and Chaplin, 2015). This discrepancy may be due to the operational definition used in the current study, which emphasizes “academic” rather than “emotional” involvement. Academic involvement—such as supervising homework or test performance—may not convey warmth or security in the same way that emotional responsiveness does. Moreover, in Confucian-influenced contexts, academic involvement may even be interpreted by students as instrumental or performance-oriented rather than nurturing (Luo et al., 2023). This finding underscores the need for future studies to distinguish between different forms of parental involvement.

Furthermore, the results indicate that materialism positively predicts internalizing problem behaviors, a relationship consistently supported by the literature. Adolescents with strong materialistic values tend to report lower psychological well-being, greater depressive symptoms, and higher anxiety (Belk, 1985; Kasser and Ahuvia, 2002; Richins, 1995; Sirgy, 1998). Kasser and Ahuvia (2002) argued that prioritizing material goals can crowd out intrinsic needs such as relatedness and autonomy, increasing psychological distress. More recent work also suggests that materialism is associated with dysregulated reward sensitivity and overactivation of the ventral striatum, contributing to emotion-driven coping and avoidance behaviors (Kim and Lee, 2024). Thus, the pathway from parental academic stress → materialism → internalizing behaviors may reflect a maladaptive coping process centered on external validation.

Comparatively, psychological resilience exhibits a stronger mediating effect than materialism (14%), accounting for 25% of the total indirect effect (Table 3), whereas materialism accounts for 22%. This suggests that positive psychological resources offer more robust protection against internalizing symptoms than avoidance-based coping strategies. According to the Conservation of Resources (COR) theory (Hobfoll, 2002), individuals with greater internal resources, such as resilience, are more likely to mobilize further resources and adapt effectively. Parental support strengthens resilience, promoting emotional regulation and buffering academic pressure, while materialism—rooted in external validation and impulsive coping—may offer only transient relief. This distinction is further supported by Adler and Stewart’s (2010) socio-psychobiological integration model, which posits that the social environment (e.g., family) affects mental health via individual-level resources and vulnerabilities. Neurocognitive research corroborates this divergence: resilience is associated with top-down regulatory systems (e.g., prefrontal cortex) that facilitate flexible reappraisal and self-control (Wu et al., 2023), while materialism activates subcortical reward circuits (e.g., nucleus accumbens), reflecting stress-induced reactive behavior (Kim and Lee, 2024). These distinct pathways may explain the observed difference in mediation strength between resilience and materialism.

The combined mediating effect of resilience and materialism accounts for 39% of the total effect between perceived parental academic stress and internalizing problem behaviors. Compared with prior studies, this proportion indicates a substantial mediation. For example, a study by Peng et al. (2023) found that emotional regulation mediated 28% of the relationship between parental control and adolescent anxiety, while Zhang and Liu (2024) reported that coping style mediated 33% of the effect of school pressure on depression symptoms. Thus, the current study’s 39% total mediation effect demonstrates relatively strong explanatory power and suggests that resilience and materialism are critical pathways worth targeting in interventions.

### 4.4 Research significance, limitations, and future directions

This study offers incremental theoretical and practical insights into the mental health of adolescents in international school settings in Mainland China—a population that remains underrepresented in psychological and educational research. By simultaneously examining perceived parental academic involvement and stress, the study provides a more comprehensive understanding of how both supportive and pressuring parenting practices relate to adolescents’ internalizing problem behaviors. This dual perspective responds to prior research that has often focused on either support (Jeynes, 2012; Graves and Wright, 2011) or pressure (Quach et al., 2015; Lee, 2006) in isolation. Moreover, the study highlights psychological resilience and materialism as distinct mediating mechanisms through which parental academic socialization affects adolescents’ emotional well-being. While previous studies have linked resilience with academic adjustment (Aysel et al., 2019), few have examined its role in buffering the emotional consequences of perceived parental academic stress. The incorporation of materialism as a mediating variable adds an additional layer by considering externally oriented, reactive coping mechanisms, expanding on recent neurocognitive findings (Kim and Lee, 2024). Together, these elements contribute to a more nuanced framework for understanding how family-based academic socialization strategies shape adolescent emotional outcomes in high-pressure educational environments, particularly within Confucian-influenced cultural contexts (Zhang et al., 2023).

Importantly, the study also reveals that perceived parental involvement did not significantly predict materialism—a non-significant finding that warrants further investigation. This result may suggest that adolescents do not necessarily equate involvement with pressure or consumer-driven coping, particularly when parental involvement is perceived as supportive rather than controlling. It underscores the need to differentiate the quality and tone of parental involvement in future research.

Several limitations should be noted. First, the study focused specifically on parental academic involvement and pressure, without incorporating other important dimensions such as emotional support, parental monitoring, or family leisure activities. Broader family dynamics and stressors beyond academics, including peer and societal pressures, were not assessed but may play a role in adolescent mental health. Second, the sample was drawn from a single international school in Beijing, which limits the generalizability of the findings. Comparative research across diverse school types, cities, and national contexts—especially through cross-cultural or multi-site designs—would enhance the robustness of the conclusions. Third, all data were self-reported and collected from adolescents, raising the possibility of common method variance. Multi-informant approaches incorporating parent or teacher reports and objective behavioral data could strengthen future analyses. Fourth, the cross-sectional nature of the data limits the ability to infer causal relationships. Longitudinal or experimental designs are needed to verify the temporal ordering of the observed pathways and test intervention effects over time.

In sum, this research contributes to a growing understanding of how academic socialization within families interacts with adolescents’ internal coping mechanisms and emotional well-being. By shedding light on both protective (resilience) and risk-enhancing (materialism) processes, it offers theoretical and practical guidance for promoting healthier developmental outcomes among youth in academically demanding environments.

## 5 Practical and clinical implications

This study yields several actionable insights for educational and clinical interventions aimed at reducing internalizing problem behaviors among adolescents. First, balanced parental involvement—characterized by warmth, open communication, and appropriate academic support without excessive control—has been shown to reduce psychological distress. Schools should guide parents through workshops or counseling sessions to shift from performance-driven parenting to support-based engagement. Second, given the key mediating role of psychological resilience, interventions should prioritize resilience-building programs in both family and school contexts. Evidence-based programs such as cognitive-behavioral therapy (CBT)-informed resilience training and school-based social–emotional learning (SEL) curricula have demonstrated effectiveness in reducing anxiety and depression by enhancing adolescents’ coping skills (Nguyen et al., 2023; Zhang and Fan, 2025). Schools could implement structured group sessions, peer mentoring, or strength-based classroom activities to foster adaptive responses to stress. Third, the findings underscore the need to de-emphasize materialistic values, which are linked to elevated emotional distress. Parents and educators should model and reinforce values such as self-efficacy, interpersonal connection, and intrinsic motivation through daily communication, media literacy education, and experiential learning (e.g., volunteering, cultural exchange). Adolescents should be taught to recognize the emotional costs of materialism and develop healthy consumption habits and delayed gratification strategies. Together, these approaches offer a roadmap for cultivating psychological strengths and protective family dynamics, particularly in high-pressure academic environments. By integrating these findings into prevention and intervention strategies, schools and families can promote adolescents’ mental health and holistic development.

## 6 Conclusion

The findings from this study reveal distinct patterns in how adolescents attending international schools in Mainland China are affected by their perceptions of parental behavior. Adolescents who perceive higher levels of supportive parental involvement report fewer symptoms of anxiety and depression, an effect that is partially explained by enhanced psychological resilience. In contrast, adolescents who perceive higher parental academic stress report greater internalizing problem behaviors, and this relationship is significantly mediated by both reduced resilience and increased materialism. Notably, while resilience serves as a protective factor in both models, materialism functions as a stress-related risk factor only in the pathway from parental stress—not involvement—to internalizing symptoms. The pathway from perceived parental involvement to internalizing problems via materialism was non-significant, suggesting that involvement alone does not foster materialistic tendencies. These results highlight the distinct psychological mechanisms through which parental support versus pressure may influence adolescents’ emotional well-being.

## Data Availability

The original contributions presented in the study are included in the article/Supplementary material, further inquiries can be directed to the corresponding author.
